# EXPLORING THE BIOLOGICAL AGING DYNAMICS IN A RENAL DEVELOPMENT ORGANOID MODEL

**DOI:** 10.1093/geroni/igad104.2488

**Published:** 2023-12-21

**Authors:** Tomoko Kasahara, Bohan Zhang, Alexander Tyshkovskiy, Yoshiyasu Tongu, Takaaki Abe, Vadim Gladyshev

**Affiliations:** Tohoku University Graduate School of Medicine, Sendai, Miyagi, Japan; Brigham and Women’s Hospital, Boston, Massachusetts, United States; Brigham and Women’s Hospital and Harvard Medical School, Boston, Massachusetts, United States; Tohoku University, Sendai, Miyagi, Japan; Tohoku University, Sendai, Miyagi, Japan; Harvard Medical School, Boston, Massachusetts, United States

## Abstract

Aging clocks emerge have emerged as promising molecular estimators of biological age based on DNA methylation or transcriptomic profiles. Among other insights, they revealed “ground zero” of aging during mouse embryogenesis characterized by the lowest biological age. To model the biological age dynamics during human embryogenesis, we established a differentiation culture system that mimics embryonic kidney development from human iPSCs. We then applied multiple aging clocks to the samples from hiPSC to kidney organoid stages. This analyses revealed the lowest biological age around the intermediate mesoderm stage, indicating a potential rejuvenation event at an early stage of renal development. A principal component analysis suggested that development in this system may comprise two processes with distinct contributions to aging, with one process increasing monotonically during development, while the other increasing at primitive streak and decreasing after the mesoderm phase. We further examined the relationship between these two processes and our previously-established transcriptomic signatures of aging and reprogramming. Interestingly, the former process may represent mostly age-related genes, while the latter process reprogramming-related genes. These findings suggest the occurrence of “ground zero” in a cell culture model of early embryonic development and characterize it in the context of molecular pathways and transcriptomic signatures.

